# Bayesian Estimation of the Active Concentration and Affinity Constants Using Surface Plasmon Resonance Technology

**DOI:** 10.1371/journal.pone.0130812

**Published:** 2015-06-22

**Authors:** Feng Feng, Thomas B. Kepler

**Affiliations:** 1 Department of Microbiology, Boston University School of Medicine, Boston, Massachusetts, 02118, United States of America; 2 Department of Mathematics & Statistics, Boston University, Boston, Massachusetts, 02118, United States of America; Irvine, UNITED STATES

## Abstract

Surface plasmon resonance (SPR) has previously been employed to measure the active concentration of analyte in addition to the kinetic rate constants in molecular binding reactions. Those approaches, however, have a few restrictions. In this work, a Bayesian approach is developed to determine both active concentration and affinity constants using SPR technology. With the appropriate prior probabilities on the parameters and a derived likelihood function, a Markov Chain Monte Carlo (MCMC) algorithm is applied to compute the posterior probability densities of both the active concentration and kinetic rate constants based on the collected SPR data. Compared with previous approaches, ours exploits information from the duration of the process in its entirety, including both association and dissociation phases, under partial mass transport conditions; do not depend on calibration data; multiple injections of analyte at varying flow rates are not necessary. Finally the method is validated by analyzing both simulated and experimental datasets. A software package implementing our approach is developed with a user-friendly interface and made freely available.

## Introduction

Surface plasmon resonance (SPR) was first applied to the study of molecular binding reactions in biology and chemistry in the 1980s [1–3]. The first commercial SPR instrument was introduced by BIACore in 1991 [4, 5]. Since then, instruments using SPR have gained increasing popularity due to their high sensitivity and simple construction, and have become the accepted standard for measuring the kinetic rate constants for molecular binding interactions. SPR technology has many practical advantages: 1) it allows real-time detection of binding events; 2) no labeling is required; 3) it can generally be applied to binding reactions of many types, such as protein-protein, protein-peptide, protein-DNA, and protein-small molecule; 4) analysis can be carried out on colored or turbid samples without interference from absorption and scattering.

Since SPR measures function—binding between analyte and ligand—it is able to determine active concentration, which is not necessarily identical to the total concentration as measured, for example by optical density. Two approaches have been proposed. The more widely used approach is the “calibration-dependent” method [6–8]. Each quantification run, however, requires a new calibration curve, thereby increasing the time and total material cost. The other approach does not rely on explicit calibration. Christensen [9] developed the mathematical theory and computed the analytical solutions under the partial mass transport conditions for molecular interactions within a two-compartment model. This approach has proven to be useful in applications [8, 10]. The method, however, has a few restrictions. It only exploits the initial binding phase of the sensorgram, requires several injections of analyte at various flow rates for each unknown sample, and assumes low noise levels on response signals in order to accurately determine the rates of change. These restrictions limit the applicability of this method. Sigmundsson *et al*. in 2005 [11] proposed a more general solution for the same process, and derived a complete analytical solution over the entire binding phase [9]. The solution involves Lambert’s *W* function, a special function that is not available in many common statistical software packages. Moreover, no user-friendly software implementation has become available as a result of either effort.

In this work, we develop a Bayesian approach to estimating the active concentration and kinetic rate constants from SPR experimental data using the same two-compartment model and quasi-steady state approximation used by Christensen and Sigmundsson [9, 11]. Bayesian statistical inference has been widely used in many biomedical applications and has been proved useful for accounting for multiple sources of uncertainty arising from experimental variation, instrumental noise, etc. [12–16]. In addition, it does not require point estimation or linearization of variations, either of which can lead to inconsistency in the estimation in nonlinear models [17, 18]. Inference of model parameters is given by the posterior density conditional on observed data. Here, we supply prior probabilities on the parameters and derive a likelihood function which together with the SPR data themselves allow us to compute the posterior probability densities on the active concentration and kinetic rate constants. This approach exploits information from the duration of the process in its entirety, including both association and dissociation phases, under partial mass transport conditions. Compared with the two approaches mentioned above, ours does not need multiple injections of analyte at varying flow rates as long as the partial mass transport conditions are met. Finally our methods have been implemented in software and are freely available.

## Theory

The theory for molecular binding under conditions of partial mass transport in a two-compartment model was worked out by Christenson [9] and is briefly recapitulated here. If *A*_0_ is the analyte in the bulk flow, *A*_*s*_ is the analyte on the biosensor surface, *B* is the ligand immobilized on the surface and *AB* is the analyte-ligand complex, the binding process in the SPR biosensor can be described by the subprocesses,

$$A_{0}\overset{\;k_{M}\;}{\underset{\;k_{M}\;}{⇌}}A_{S}$$ (1)

and

$$A_{S}+B\overset{\;k_{a}\;}{\underset{\;k_{d}\;}{⇌}}AB$$ (2)

where *k*_*M*_ is the mass transport coefficient, and *k*_*a*_ and *k*_*d*_ are the association and dissociation rate constants, respectively. Process (1) describes the mass transport of the analyte between the bulk flow (*A*_*0*_) and surface (*A*_*s*_); process (2) describes the binding and unbinding of analyte and ligand on the surface matrix.

The mass action equations for these processes are

$$h_{diff}\frac{d\left[A_{S}\right]}{dt}=k_{M}\left(\left[A_{0}\right]-\left[A_{S}\right]\right)-k_{a}\left[A_{S}\right]\left[B\right]+k_{d}\left[AB\right]$$ (3)

and

$$\frac{d[AB]}{dt}=k_{a}\left[A_{S}\right]\left[B\right]-k_{d}[AB]$$ (4)

where *h*_*diff*_ is the characteristic height of the diffusion layer, given [19] approximately by

$$h_{diff}\approx \sqrt[{3}]{\frac{Dh^{2}wl}{F}}$$ (5)

*D* is the analyte diffusion coefficient, *F* is the bulk flow rate and *h*, *w* and *l* are the height, width and length of the flow chamber of the SPR system, respectively [19]. The diffusion coefficient *D* can be estimated by Stoke’s law and Einstein-Sutherland equation [20] (See details in S1 Appendix).

Under the quasi-steady state condition (where *d*[*As*]/*dt = 0* in Eq (3) is assumed) [21], the analyte concentration at the biosensor surface matrix can be approximated by:

$$[A_{s}]=\frac{k_{M}[A_{0}]+k_{d}[AB]}{k_{M}+k_{a}[B]}$$ (6)

which describes the average of the concentration gradient that forms along the surface [21]. Combining Eqs (4) and (6)and rearranging give

$$\frac{d[AB]}{dt}=\frac{k_{a}[A_{0}][B]-k_{d}[AB]}{1+k_{a}[B]/k_{M}}$$ (7)

which has been evaluated and demonstrated to be a reasonable approximation for most conditions with the partial mass transport effect [9, 11]. Under experimental conditions, the analyte bulk concentration [*A*_0_] remains constant by a continuous injection of fresh analyte solution at a fixed flow rate. The amount of free ligand [*B*] decreases with time until steady-state is reached. Furthermore, the density of free ligand can be expressed through the conservation relation as

$$\left[B]=[B_{\text{max}}]-[AB\right]$$ (8)

where [*B*_*max*_] is the density of total ligand (bound and unbound) on the surface and [*AB*] is the density of bound complex.

The mass transport coefficient, *k*_*M*_, is approximately proportional to the cube root of the flow rate [22],

$$k_{M}=C_{k_{M}}\sqrt[{3}]{\frac{D^{2}F}{h^{2}wl_{2}}}$$ (9)

and

$$C_{k_{M}}=1.47\frac{1-{\left(l_{1}/l_{2}\right)}^{2/3}}{1-l_{1}/l_{2}}$$ (10)

where *l*_1_ and *l*_2_ are the lengths to the start and end, respectively, of the detection area from inlet of the flow cell [22]. For a BIAcore system, these values are known. Therefore, if the molecular weight and the bulk flow rate of the analyte are also given, *k*_*M*_ can be obtained. When *l*_1_ and *l*_2_ or other parameters (such as *h*, *w* or *l*) are not easily obtained for a biosensor system other than BIAcore instruments, *k*_*M*_ can still be estimated empirically (see discussions in the Results section).

The response signal, *R*, output by an SPR sensor is proportional to the amount of complex formed at the biosensor surface with an empirical factor given by

$$R=W_{M}\;G[AB]$$ (11)

where *G* is the response per mass per area for proteins with an approximate value of 1000 RU·mm^2^/ng [23] and *W*_*M*_ is the molecular weight of analyte. Therefore, Eq (7) now can be rewritten as

$$\frac{d\left[R\right]}{dt}=\frac{k_{a}\left[A_{0}\right]\left(\left[R_{max}\right]-R\right)-k_{d}[R]}{1+k_{a}\left(\left[R_{max}\right]-R\right)/k_{M}}$$ (12)

where *R*_*max*_ is the maximum value of the response signal when all the immobilized ligands have been fully bound into complexes, and *k*_*M*_ is rescaled to have unit of *RU/M/s* instead of *m/s*.

The mass transport limitation for a specific analyte-ligand system is determined by both the amount of free immobilized ligand and the bulk flow rate of the analyte (*k*_*M*_). A limit coefficient, *k*_*a*_[*B*]*/k*_*M*_, can be obtained from Eq (7) to identify conditions for mass transport-limited binding and for kinetic binding (see S1 Appendix for details). Practically, zero and full mass transport limitation are not easily obtained with a given analyte-ligand system, since to approach these extreme conditions it is necessary to use a wide range of ligand concentrations. On the other hand, it is relatively easy to obtain partial mass transportation limitation. In the next section we describe a Bayesian approach to determining the active concentration as well as the rate constants under the partial mass transport conditions of SPR responses.

## Bayesian Inference

We use Bayesian methods to obtain the posterior distributions of active concentrations, *A*_0_, association/dissociation rate constants, *k*_*a*_ and *k*_*d*_, the theoretical maximum response signal, *R*_*max*_, and the initial response level at the beginning of the dissociation phase, *R*_*0*_. As discussed in the previous section, the analyses are performed on SPR data assuming partial mass transport limitation conditions described by the differential equation Eq (12). The initial-value problem for Eq (12) does not have a closed-form solution, therefore we carry out inference on its numerical solution.

In addition to the process model, we must have a measurement, or statistical, model. The statistical model for the *i*th SPR sensorgram signal *R*_*i*_ conditional on the parameter vector *θ* ≡ {*A*_0_, *k*_*a*_, *k*_*d*_,…} is

$$R_{i}=r\left(t_{i},\theta \right)+\varepsilon_{i}$$ (13)

where the errors *ε*_*i*_ are independent, identically-distributed Gaussian random variables with mean zero and variance *σ* ^2^.

$$\varepsilon_{i}∼N(0,\sigma^{2})$$ (14)

The following diffuse prior distributions are chosen [24, 25].

$$k_{a}∼N(5\times 10^{11},10^{12})$$ (15)

$$k_{d}∼N(50,10^{2})$$ (16)

$$R_{\text{max}},R_{0}∼N(5\times 10^{4},10^{5})$$ (17)

$$\sigma^{2}∼InvGamma(0.1,0.1)$$ (18)

The variances for the normal distributions are set so to make the prior distributions sufficiently diffuse/non-informative. Given the amount of observed data in a typical SPR experiments, the posterior distributions of these parameters are insensitive to modest variation in the prior distributions.

For estimation of the posterior density, we use the Markov Chain Monte Carlo (MCMC) method with the Gibbs sampler to generate samples from the posterior distributions of parameters as follows.

- Define the full conditional distributions [26] as
- {*π*(*θ*_1_ | *θ*_2_, *θ*_3_, .... *θ*_*p*_); *π*(*θ*_2_ | *θ*_1_, *θ*_3_, .... *θ*_*p*_); ..... *π*(*θ*_*p*_ | *θ*_1_, *θ*_2_, .... *θ*_*p*−1_)}

One cycle of the Gibbs sampler is completed by drawing ${\left\{\theta_{k}\right\}}_{k=1}^{p}$ from these distributions successively updating the conditional variables

Algorithm

1. define the initial values, $\theta^{\left(0\right)}=\left(\theta_{1}^{\left(0\right)},\;\theta_{2}^{\left(0\right)},\;\theta_{3}^{\left(0\right)},\;\ldots .\;\theta_{p}^{\left(0\right)}\right)$;
2. repeat for j = 1.....M
  - Generate $\theta_{1}^{\left(j+1\right)}$ from $\pi \left(\theta_{1}|\;\theta_{2}^{\left(j\right)},\;\theta_{3}^{\left(j\right)},\;\ldots .\;\theta_{p}^{\left(j\right)}\right)$;
  - Generate $\theta_{2}^{\left(j+1\right)}$ from $\pi \left(\theta_{2}|\;\theta_{1}^{\left(j+1\right)},\;\theta_{3}^{\left(j\right)},\;\ldots .\;\theta_{p}^{\left(j\right)}\right)$;
  - ……
  - Generate $\theta_{p}^{\;(j+1)}$ from $\pi \left(\theta_{p}|\;\theta_{1}^{\left(j+1\right)},\;\theta_{2}^{\left(j+1\right)},\;\ldots .\;\theta_{p-1}^{\;(j+1)}\right)$;
3. return values {*θ* ^(1)^, *θ* ^(2)^, *θ* ^(3)^, .... *θ* ^(*M*)^}

Following an equilibration period, the Markov chain approaches its stationary distribution and samples from the MCMC closely approximate samples from the posterior parameter distribution. Using these samples, the posterior means and independent 95% Bayesian credible intervals (BCI) from the posterior marginal densities are computed.

Combining the priors and observed data, the full conditional probability can be formulated as

$$k_{a}:\pi ({\text{k}}_{a}|k_{d},A_{0},R_{\text{max}},R_{0},\sigma^{2},R_{t},t)\propto \prod_{i}e^{-\frac{{\left(R_{t}-r(t,\theta )\right)}^{2}}{2\sigma^{2}}}\times e^{-\frac{{\left(k_{a}-5\times 10^{11}\right)}^{2}}{2\times 10^{12}}}$$ (19)

$$k_{d}:\pi ({\text{k}}_{d}|k_{a},A_{0},R_{\text{max}},R_{0},\sigma^{2},R_{t},t)\propto \prod_{i}e^{-\frac{{\left(R_{t}-r(t,\theta )\right)}^{2}}{2\sigma^{2}}}\times e^{-\frac{{\left(k_{d}-50\right)}^{2}}{2\times 10^{2}}}$$ (20)

$$A_{0}:\pi (A_{0}|k_{a},k_{d},R_{\text{max}},R_{0},\sigma^{2},R_{t},t)\propto \prod_{i}e^{-\frac{{\left(R_{t}-r(t,\theta )\right)}^{2}}{2\sigma^{2}}}\times e^{-\frac{{\left(A_{0}-50\right)}^{2}}{2\times 10^{2}}}$$ (21)

$$R_{\text{max}}:\pi (R_{\text{max}}|k_{a},k_{d},A_{0},R_{0},\sigma^{2},R_{t},t)\propto \prod_{i}e^{-\frac{{\left(R_{t}-r(t,\theta )\right)}^{2}}{2\sigma^{2}}}\times e^{-\frac{{\left(R\text{max}-5\times 10^{4}\right)}^{2}}{2\times 10^{5}}}$$ (22)

$$R_{0}:\pi (R_{0}|k_{a},k_{d},A_{0},R_{\text{max}},\sigma^{2},R_{t},t)\propto \prod_{i}e^{-\frac{{\left(R_{t}-r(t,\theta )\right)}^{2}}{2\sigma^{2}}}\times e^{-\frac{{\left(R_{0}-5\times 10^{4}\right)}^{2}}{2\times 10^{5}}}$$ (23)

$$\sigma^{2}:\pi (\sigma^{2}|k_{a},k_{d},A_{0},R_{\text{max}},R_{0},R_{t},t)\propto \prod_{i}e^{-\frac{{\left(R_{t}-r(t,\theta )\right)}^{2}}{2\sigma^{2}}}\times e^{-\frac{0.1}{2\sigma^{2}}}\times \sigma^{-n-2.2}$$ (24)

**Fig 1:**
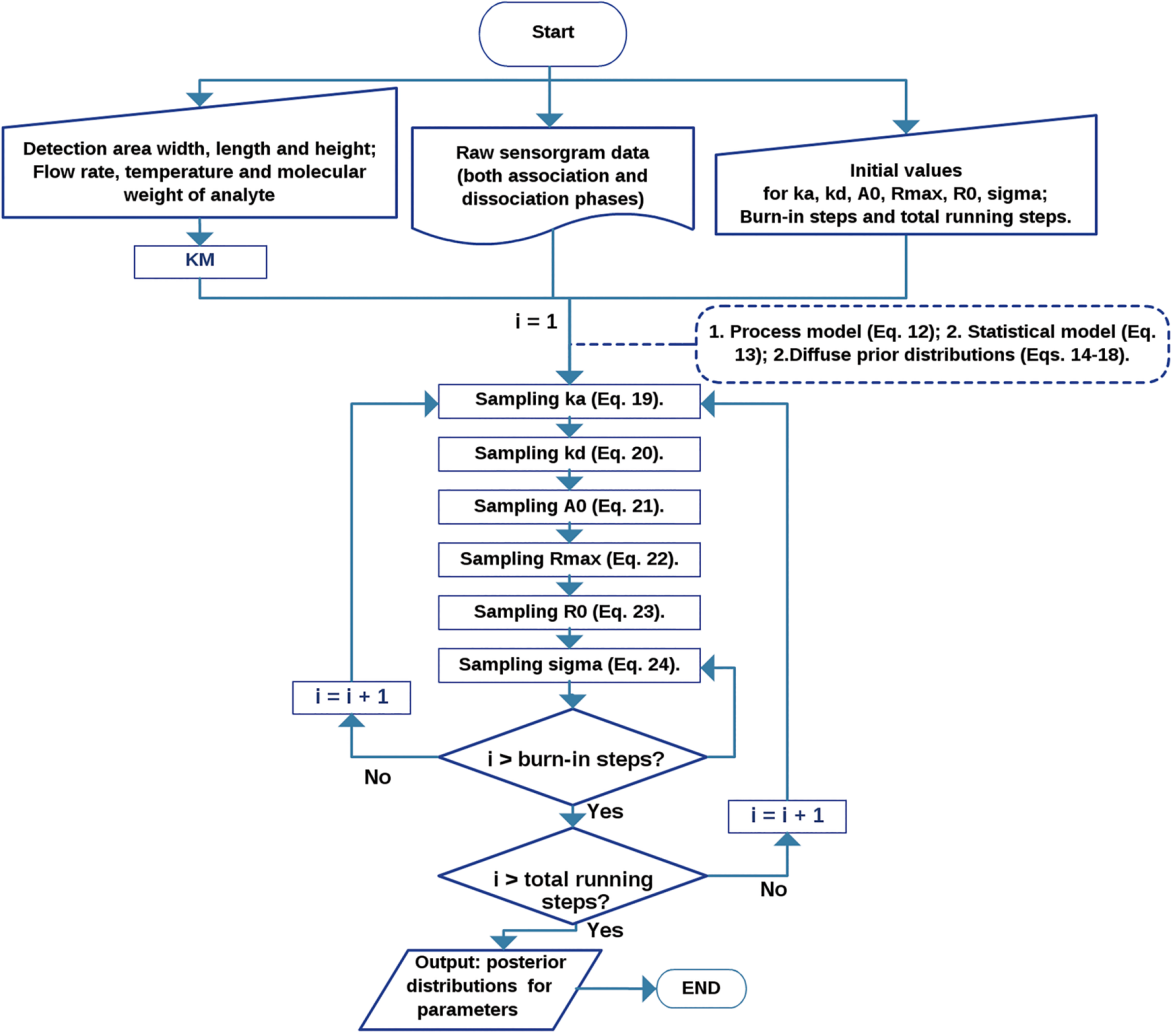
Flow chart summarizing the inference processes in our proposed Bayesian approach.

Because we are using the numerical solution to a dynamical system initial value problem, we employ the adaptive rejection with Metropolis sampling (ARMS) algorithm [27, 28] with modifications [29], which allows one to draw samples from an arbitrary distribution. The overall inference processes have been summarized in Fig 1 flow chart.

## Software Development

**Fig 2:**
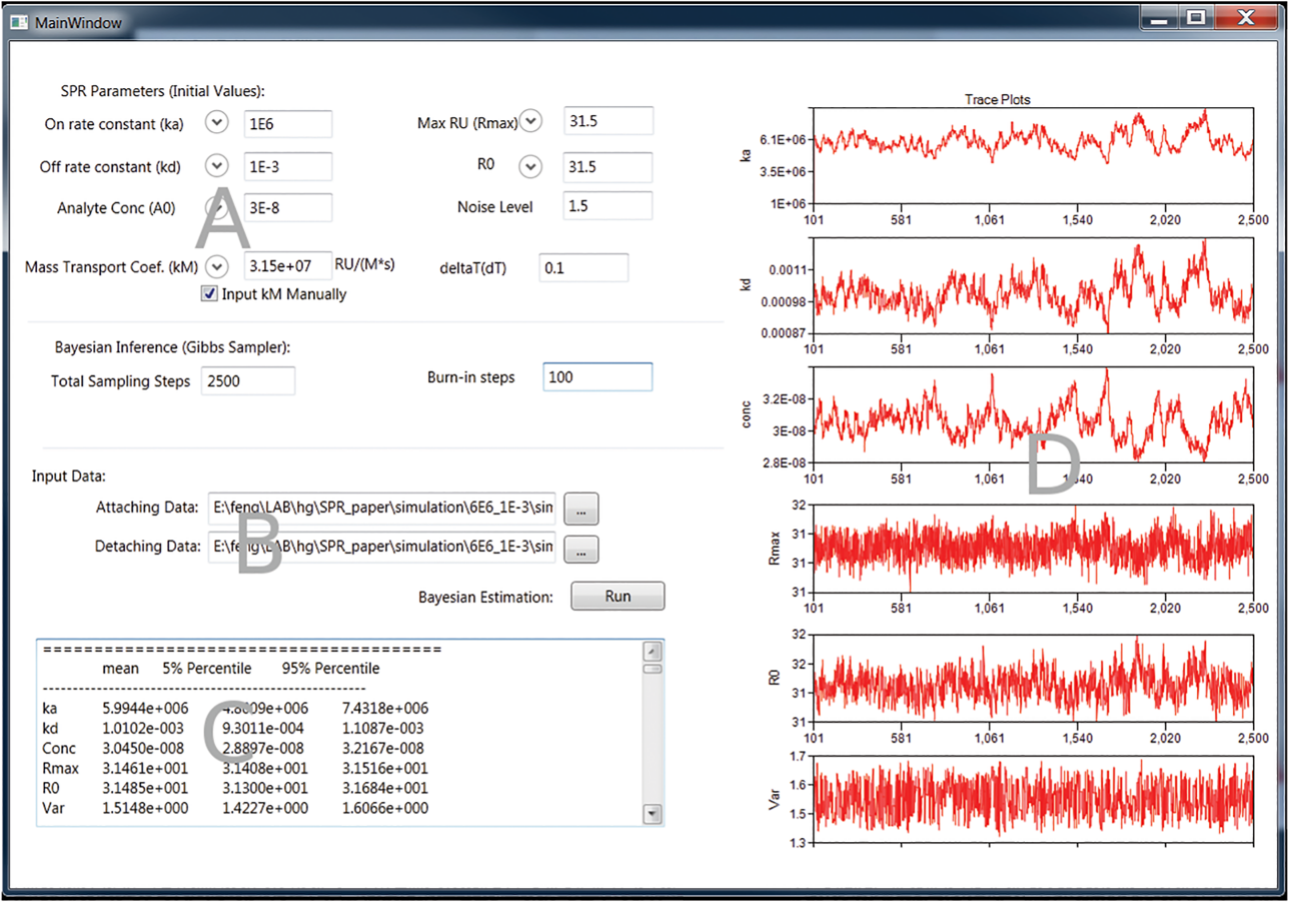
Screen Shot of the implemented software. The implemented software has the interface to allow the user to define the initial values of the parameters (A) and specify the input files (B) as well as controlling the behavior of the Gibbs sampler inference. It also has the interface to show the summary statistics (C) and trace plots of estimations (D).

The software implementation of the proposed approach was developed in C# for the .NET framework. The executable is available for free distribution. The program has a simple and intuitive graphical interface. Fig 2 shows a screenshot of the interface and illustrates its various components. The input of the program is through text data files. Users can specify the initial values of the parameters through the interface. The results are then written to files with simulated posterior distributions, allowing further analysis such as MCMC diagnostics, hypothesis testing and the fitting of models for biological processes.

Because the proposed approach requires numerical solution of the initial value problem for differential equation Eq (12), the analysis is a computation-intensive task. Special optimization algorithms have been incorporated to make the software efficient, e.g. the forth-order Runge-Kutta algorithm for calculating numerical solution and the Nelder-Mead algorithm for finding maxima and minima of functions.

## Experimental Procedures

### Reagents

Cabonic anhydrase isozyme II (CAII) (MW = 30kD) from bovine erythrocytes, 4-carboxybenzenesulfonamide (4-CBS) (MW = 201.2D) and TWEEN 20 were purchased from Sigma (St. Louis, MO). N-(3-dimethylaminopropyl-N’-ethylcarbodiimide (EDC), N-hydroxysuccinimide (NHS), ethanolamine-HCl and sodium acetate were purchased from Bio-Rad (Hercules, CA).

### Surface plasmon resonance measurements

SPR measurements were carried out on a SensiQ Pioneer biosensor (SensiQ Technologies, Inc., OK). The CHOO2 two dimensional surface chip was used in the assay. The ligand CAII was immobilized via the common amine coupling chemistry. This involved activating the chip surface with freshly made 2mM EDC and 0.5mM NHS (as suggested by the manufacturer), injecting CAII in sodium acetate (pH 5.0) and then blocking excess reactive esters with ethanolamine. Subsequently, the analyte 4-CBS was injected with a concentration of 50μM at a flow rate of 50μl/min for 60 seconds and followed with a dissociation phase for another 60 seconds. The interaction was carried out in PBS with 0.05% TWEEN 20 (pH 7.0) (PBST) at 25˚C.

### Data analysis

The SPR response data were first processed in SensiQ Qdat (version 2.2) software for background subtraction, and then were exported as text files for analyses using the software development in this work.

## Results

We validated the method using simulated SPR response data, experimental SPR data generated in our laboratory, and publicly available experimental data.

### Validation on Simulated Data

**Fig 3:**
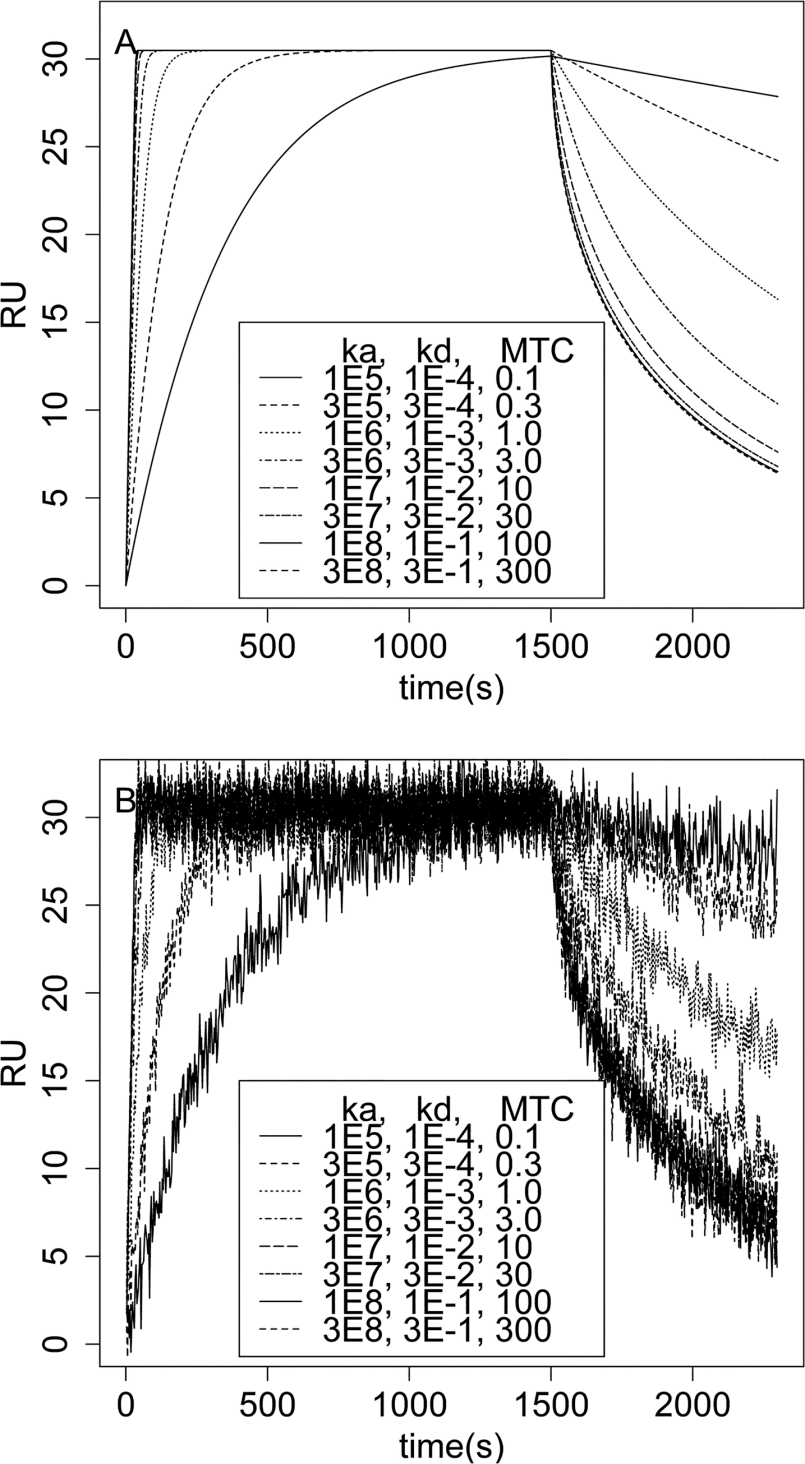
Simulated SPR response data. (A), the SPR response data were simulated according to Eqs (12), (13) and (14) with no measurement error. In simulations *R*_*max*_ was 31.5 RU, *A*_0_ was 30nM, *k*_*a*_ values varied between 1x10^5^ and 3x10^8^/M/s, *k*_*d*_ values were adjusted so that *K*_*D*_ was constant 1nM and *k*_*M*_ was 3.15x10^7^ RU/M/s. The mass transport limiting coefficients (MTLC) of these responses varied between 0.1 (little/no mass transport effect) and 300 (high/full mass transport effect). (B), same as in A, but measurement error with a constant standard deviation of 1.5 RUs was added to the simulated data. The level of noise was chosen based on empirical data obtained from the Bio-Rad Proteon XPR36 biosensor in our lab through a variety of antibody-antigen interactions (1~3 RUs in our experiments).

The proposed Bayesian approach was validated by analyzing simulated SPR response data. To simulated SPR data, a strategy similar to the one employed by Karlsson were adopted [30]. Eq (12) was used to simulate interaction data for reactions with *k*_*M*_ = 3.15 x 10^7^ RU/M/S. This *k*_*M*_ value is similar to that in the Inogatran/Thrombin system [30]. The concentration of analyte was set to 30nM, the maximum immobilized ligand in terms of the response signal (*R*_*max*_) was 31.5RU, *k*_*a*_ values varied between 1x10^5^ and 3 x 10^8^ /M/s, *k*_*d*_ values were adjusted so that the affinity, *K*_*D*_, was held constant at 1nM. The response curves in Fig 3 represent binding curves with differing kinetic rate constants, but constant *K*_*D*_. The Gaussian noise at a level of 1.5RUs, which was equivalent to that (1~3RUs) seen on Bio-Rad Proteon XPR36 biosensors but bigger than that on BIACORE and SensiQ systems, was added to the simulated data as specified by Eqs (13) and (14).

**Fig 4:**
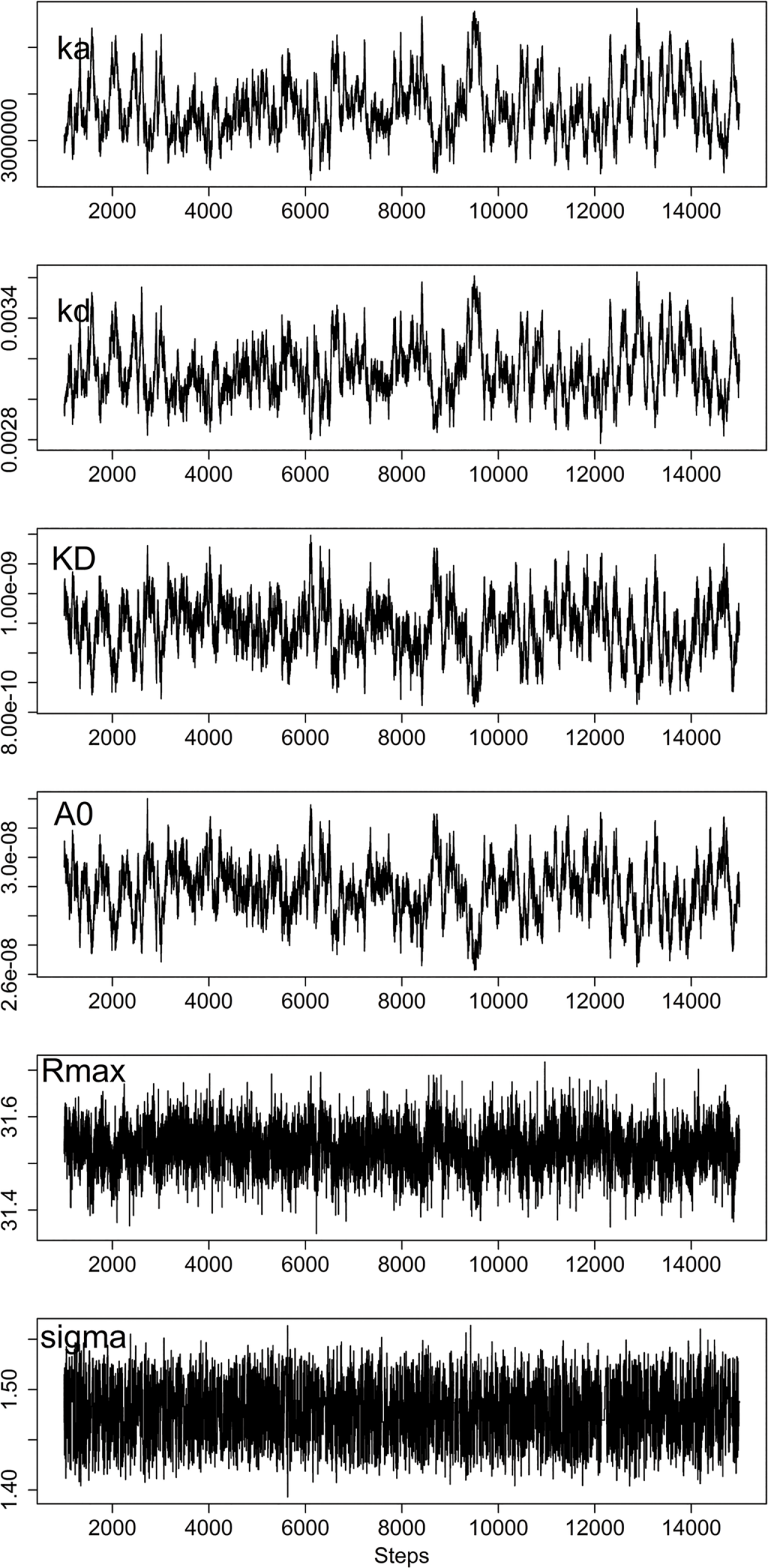
The trace plots for evaluation of the simulated SPR response data. The SPR response data were simulated as in Fig 2 with *R*_*max*_ 31.5RU, *k*_*a*_ 3x10^6^/M/s, *k*_*d*_ 3x10^-3^/s, *k*_*M*_ 3.15x10^7^RU/M/s, *A*_0_ 30nM and σ^2^ 1.5. The data were analyzed as described in the text. The trace plots are shown for six parameters in the analysis of the data set with *k*_*a*_ * *R*_*max*_/*k*_*M*_ = 3.0. The returned parameters are summarized in Table 1.

The simulated data were then analyzed using the proposed Bayesian method. The parameter values returned were summarized in Table 1, and the chain trace plots for one set of data (*k*_*a*_ *R_*max*_ */k*_*M*_ = 3.0) are shown in Fig 4. The proposed approach did not return accurate *k*_*a*_, *k*_*d*_ or *A*_*0*_ under the situations of little or no mass transport effect (case i and ii, where *k*_*a*_ *R_*max*_ */k*_*M*_ <1), although *R*_*max*_ and noise level *σ* ^*2*^ could be determined correctly under the same conditions. As the mass transport effect started to dominate, *k*_*a*_, *k*_*d*_ and *A*_*0*_ as well as others *(R*_*max*_ and noise level) could all be determined accurately (cases *iii~vi*, where *k*_*a*_ *R_*max*_ */k*_*M*_ was between 1~100). In case of very high mass transport effect conditions (case vii and viii, where *k*_*a*_ *R_*max*_ */k*_*M*_ >100), *k*_*a*_ and *k*_*d*_ estimations became inaccurate while the affinity constant *K*_*D*_ and other parameters were still correctly determined. Similar behavior has been reported previously [30].

**Table 1:** Parameter values estimated by the proposed Bayesian method using the simulated SPR response Data.

		*k*_*a*_ *(1/M/s)*	*k*_*d*_ *(1/s)*	*A*_*0*_ *(nM)*	*R*_*max*_ *(RU)*	*K*_*D*_ *(nM)*	*k*_*a*_ *R_*max*_ */k*_*M*_
*Parameters in simulation*							
*I*		1.0 x 10^5^	1.0 x 10^−4^	30.0	31.5	1.0	0.1
*Ii*		3.0 x 10^5^	3.0 x 10^−4^	30.0	31.5	1.0	0.3
*iii*		1.0 x 10^6^	1.0 x 10^−3^	30.0	31.5	1.0	1.0
*iv*		3.0 x 10^6^	3.0 x 10^−3^	30.0	31.5	1.0	3.0
*V*		1.0 x 10^7^	1.0 x 10^−2^	30.0	31.5	1.0	10.0
*vi*		3.0 x 10^7^	3.0 x 10^−2^	30.0	31.5	1.0	30.0
*vii*		1.0 x 10^8^	1.0 x 10^−1^	30.0	31.5	1.0	100.0
*viii*		3.0 x 10^8^	3.0 x 10^−1^	30.0	31.5	1.0	300.0
*Parameters estimated by the proposed approach*							
*I*	1.2 x 10^5^[1.1x10^5^, 1.5x10^5^]		1.1 x 10^−4^[0.8x10^-4^, 1.3x10^-4^]	25.3[20.9, 26.3]	31.0[29.9, 31.9]	0.9[0.63, 1.06]	
*Ii*	1.6 x 10^5^[1.4x10^5^, 1.9x10^5^]		3.1 x 10^−4^[3.0x10^-4^, 3.3x10^-4^]	49.8[45.3, 56.5]	31.8[31.6, 32.0]	1.8[1.66, 2.10]	
*iii*	0.95 x 10^6^[0.8x10^6^, 1.1x10^6^]		1.0 x 10^−3^[0.9x10^-3^, 1.0x10^-3^]	31.2[27.9, 35.0]	31.5[31.3, 31.7]	1.0[0.89, 1.22]	
*iv*	3.3 x 10^6^[2.9x10^6^, 3.8x10^6^]		3.1 x 10^−3^[2.9x10^-3^, 3.4x10^-3^]	28.9[27.3, 30.4]	31.5[31.4, 31.6]	1.1[0.86, 1.20]	
*V*	1.1 x 10^7^[0.9x10^7^, 1.4x10^7^]		1.1 x 10^−2^[0.9x10^-2^, 1.3x10^-2^]	29.1[28.2, 30.2]	31.5[31.4, 31.5]	1.0[0.93, 1.03]	
*vi*	4.0 x 10^7^[2.3x10^7^, 7.8x10^7^]		3.9 x 10^−2^[2.4x10^-2^, 7.3x10^-2^]	29.6[28.7, 30.5]	31.5[31.5, 31.6]	1.0[0.94, 1.03]	
*vii*	0.7 x 10^8^[0.4x10^8^, 1.0x10^8^]		0.7 x 10^−1^[0.5x10^-1^, 1.0x10^-1^]	30.3[29.8, 30.7]	31.5[31.5, 31.6]	1.0[1.00, 1.04]	
*viii*	0.6 x 10^8^[0.2x10^8^, 1.1x10^8^]		0.6 x 10^−1^[0.3x10^-2^, 1.1x10^-2^]	30.9[30.1, 31.8]	31.5[31.5, 31.6]	1.1[1.01, 1.10]	

The SPR response data were simulated according to Eqs (12), (13) and (14) with *k*_*M*_ = 3.15x10^7^ as in Fig 1. The parameters used in the simulation were specified in the top section of the table. Then the proposed approach was applied to estimate the parameters, which were listed in the bottom section in the table with the 95% Bayesian credible intervals (in square brackets). The parameters in the simulation ranged from little mass transport limited conditions (*k*_*a*_ * *R*_*max*_/*k*_*M*_ = 0.1) to high/full mass transport limited conditions *(k*_*a*_ * *R*_*max*_/*k*_*M*_ = 300).

### Validation on experimental data from our laboratory

**Fig 5:**
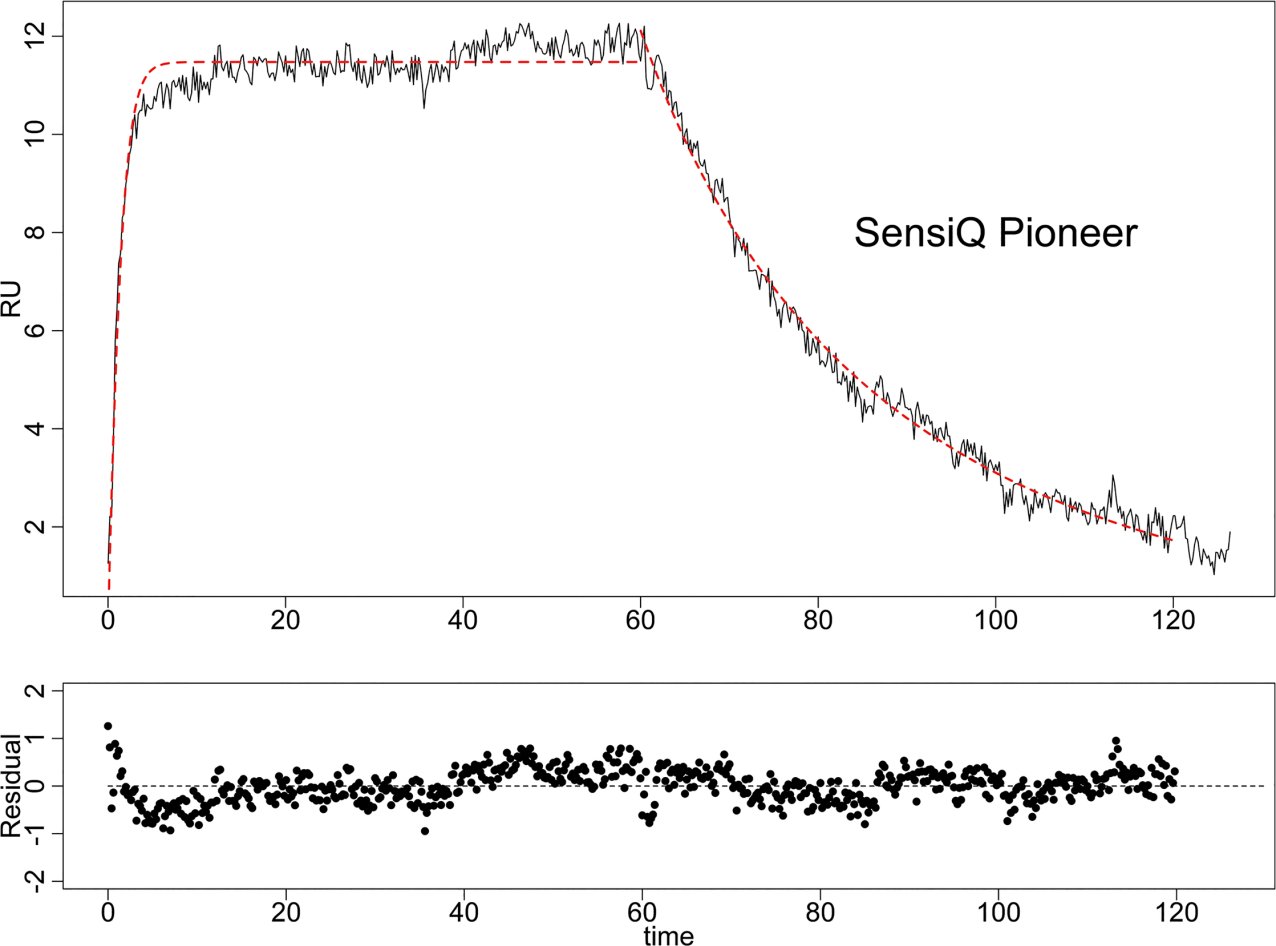
The kinetic analysis of the emzyme CAII and its small molecule inhibitor 4-CBS in the SensiQ Pioneer biosensor. CAII was immobilized by the amine coupling chemistry at a level of about 3000RU, and then 50μM 4-CBS was injected at a flow rate of 50μl/min at 25˚C. The generated sensorgram data were exported and analyzed by the proposed Bayesian approach. The diffusion coefficient and mass transport coefficient were first derived, and then the kinetic parameters as well as the active concentration were determined. The fitted values of the responses were overlaid with the original sensorgram (the red dashed line) on the top figure, and the residuals were showed on the bottom.

The proposed approach was further tested with experimental data. The enzyme carbonic anhydrase (CAII) and its small molecule inhibitor 4-carboxybenzenesulfonamide (4-CBS) were chosen to be studied on a SensiQ Pioneer biosensor. In the experiment, about 3000RU of CAII was amine coupled to the chip surface as the ligand and then 4-CBS was injected at a flow rate of 50μl/min. This experiment setting resulted in a partial mass-transport limited condition for the interaction, and generated small but detectable responses (Fig 5). The data were then exported and analyzed to determine the active concentration and the kinetic constants of the analyte. First, the mass transport coefficient *k*_*M*_, which primarily depended on the diffusion coefficient, the dimensions of the flow cell and the flow rate, had to be determined. Under the current experimental condition, the diffusion coefficient D of the analyte, 4-CBS (molecular weight = 201.2 Dalton), was calculated to be 4.76x10^-10^ m^2^/s [20] (Table A in S1 Appendix). Furthermore, the flow cell dimensions of the SensiQ Pioneer biosensor were given with a width 0.635mm, a height 0.05mm and a length 3.0mm by the manufacturer. Then with a flow rate of 50μl/min, *k*_*M*_ was determined to be 1.03x10^7^ RU/(Ms) according to Eqs (9) and (10) (Table A in S1 Appendix). Finally, the kinetic parameters and the active analyte concentration were estimated by the proposed Bayesian approach and shown in Table 2. The original sensorgram and the fitted values were overlaid in Fig 5, which showed a very good fit. The trace plots of each parameter in the analysis were shown in Fig A in S1 Appendix.

**Table 2:** Parameter values estimated by the proposed Bayesian approach using the experimental SPR response data.

	*k*_*a*_ *(1/M/s)*	*k*_*d*_ *(1/s)*	*A*_*0*_ *(nM)*	*K*_*D*_ *(nM)*
Dataset #1 (SensiQ Pioneer)				
in literature [31]	*4*.*8x10* ^*4*^	*3*.*65x10* ^*-2*^	*-*	*760*
by the proposed approach	*4*.*67x10* ^*5*^ *[3*.*66x10* ^*5*^, *5*.*84x10* ^*5*^ *]*	*4*.*2x10* ^*-2*^ *[4*.*04x10* ^*-3*^, *4*.*45x10* ^*-3*^ *]*	*2020[1705*, *2415]*	*92*.*1[76*, *110]*
Dataset #2 (Biacore 3000)				
in the reference [32]	*5*.*47x10* ^*7*^	*3*.*9x10* ^*-3*^	*1*.*25*	*0*.*0713*
by the proposed approach	*2*.*30x10* ^*8*^ *[2*.*16x10* ^*8*^, *2*.*44x10* ^*8*^ *]*	*4*.*4x10* ^*-3*^ *[4*.*30x10* ^*-3*^, *4*.*65x10* ^*-3*^ *]*	*0*.*612[0*.*61*, *0*.*62]*	*0*.*01946[0*.*0191*, *0*.*0199]*
Dataset #3 (Proteon XPR36)				
in the reference [33]	*1*.*15x10* ^*5*^	*6*.*71x10* ^*-4*^	*300*	*5*.*780*
by the proposed approach	*3*.*6x10* ^*7*^ *[2*.*7x10* ^*7*^, *4*.*7x10* ^*7*^ *]*	*8*.*2x10* ^*-4*^ *[7*.*5x10* ^*-4*^, *9*.*2x10* ^*-4*^ *]*	*13*.*73[13*.*4*, *14*.*1]*	*0*.*023[0*.*018*, *0*.*030]*

Three tests were carried out. In the first experiment, the interaction data between CAII and 4-CBS were studied on a SensiQ Pioneer biosensor under a partial mass-transport limited condition. The data were analyzed and the resulted kinetic parameters and active concentration. In the other two tests, two datasets were collected from the literature. Dataset #1 was from the work done by Castonguay *et al.[32]*, in which BMP10 interacted with immobilized human Eng^ECD^-Fc in a Biacore 3000 system. Dataset #2 was from a different work done by Abdiche *et al*.[33], in which human CGRPα bound immobilized 4901 IgG in a Proteon XPR36 biosensor. The two datasets were then analyzed by the proposed approach to estimate the active concentration of analyte *A*_0_, rate constants *k*_*a*_ and *k*_*d*_, and the dissociation constant *K*_*D*_. The returned parameters as well as the parameters estimated in the original reference papers were listed. The 95% Bayesian credible intervals for the estimation were also provided in the square brackets. The biosensor used to collect the experimental data was indicated in the parentheses.

The active concentration *A*_0_ of the analyte was determined in our analysis to be 2.0 ± 0.22 μM, which was about 4% of the total concentration (50μM of the analyte). This percentage of the active concentration in the total concentration was similar to that of the active phosphorylated GST-CEACAM1-4L protein determined by both SPR and other radioactivity based method [11]. Moreover, our analysis showed that the dissociation rate constant *k*_*d*_ was (4.23 ± 0.12)×10^−2^ *s* ^−1^, which is consistent with what has been reported in the literature ((3.65 ± 0.06)×10^−2^ s^−1^) [31]. On the other hand, the estimated association rate constant *k*_*a*_, (4.67 ± 0.66)×10^5^ *M* ^−1^ *s* ^−1^, was about ten times higher than what was reported ((4.8 ± 0.2)×10^4^ M^−1^s^−1^) in an analysis that did not take into account the difference between active concentration and total concentration [31]. Thus, the discrepancy is not a surprise since the active concentration was more than ten times lower than the total concentration. When we used the total concentration of analyte (50μM) rather than the active concentration, we get a value for *k*_*a*_ (1.4×10^4^ M^−1^s^−1^) that was similar to the previously-reported value. We argue that it is as more appropriate to use the active concentration under these circumstances, and that this comparison illustrates the importance of doing so.

### Validation on publicly available data

In order to further validate the proposed approach, we analyzed two publicly available experimental data sets generated using other popular biosensors. The first dataset was from Castonguay *et al*.[32], in which the bone morphogenetic protein 10 (BMP10) interacted with immobilized human endoglin extracellular domain-Fc chimera (hEng^ECD^-FC) in a Biacore 3000 system. The second dataset was generated on a Proteon XPR36 biosensor from the work of Abdiche *et al*.[33], in which human calcitonin gene-related peptide-α (CGRPα) reacted with immobilized 4901 IgG. These two datasets were selected because they were generated under partial mass transport-limited conditions and the molecular weights of the analytes in them were known. With the latter information, the diffusion coefficient and then the mass transport coefficient could be derived from Eqs (5), (9) and (10) (also see details in S1 Appendix about the diffusion coefficient *D*). Therefore, the proposed approach could be applied to estimate the active concentration of analyte *A*_0_ and rate constants *k*_*a*_ and *k*_*d*_. The results are listed in Table 2, together with parameter estimates from the original reference papers for comparison.

**Fig 6:**
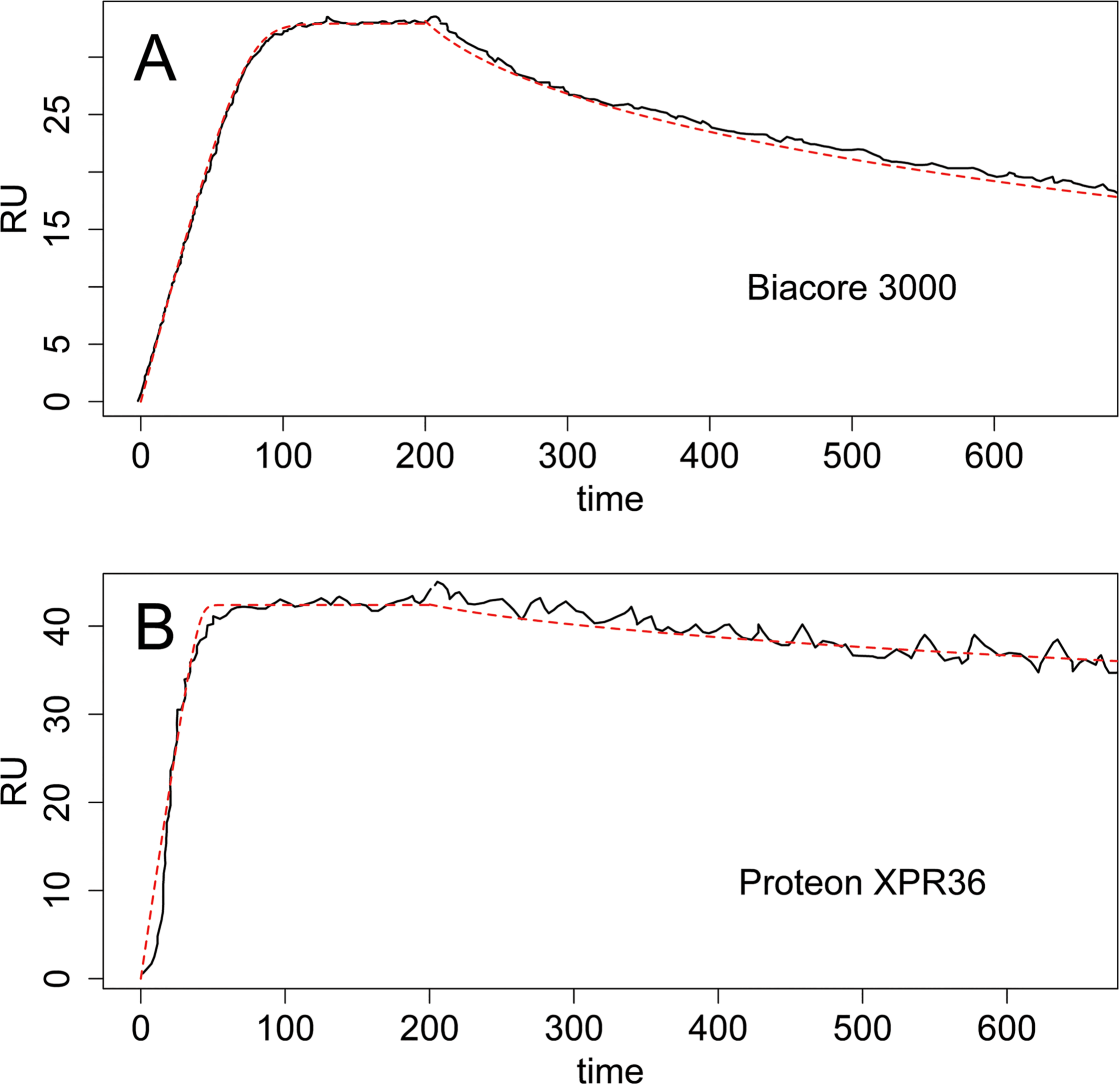
The kinetic analysis of BMP10 with hEnd^ECD^-hFC in BiaCore 3000 and hCGRPα with 4901 IgG in Proteon XPR36 by the proposed Bayesian approach. Two sets of data were extracted from literature. (A), the interaction between BMP10 and immobilized hEngECD-hFc was measured in a Biacore 3000 system [32]. (B), the analyte human CGRPα and immobilized 4901 IgG were studied in a Proteon XPR36 biosensor [33]. The values of mass transport coefficient *k*_*M*_ were derived according to Eqs (9) and (10) for both cases. The two datasets were then analyzed by the proposed approach to estimate the active concentration of analyte *A*_0_, rate constants *k*_*a*_ and *k*_*d*_, and the dissociation constant *K*_*D*_. The returned parameters were listed in Table 2. The raw sensorgrams (solid lines) and fitted curves (dashed lines) were overlaid.

In the first case, the SPR response data between BMP10 and hEng^ECD^-hFC were generated on a Biacore 3000 system, in which the detection area of the flow chamber has been very well documented. The parameters, such as the width, length and height of the measured SPR, are known [9, 11] (Table A in S1 Appendix). The diffusion coefficient was approximated using the available molecular weight of the analyte BMP10 (see details in S1 Appendix). The mass transport coefficient *k*_*M*_ was thus determined to be approximately 8x10^8^ RU/M/s (Table A in S1 Appendix) and used to estimate the active analyte concentration and the kinetic rate constants (Table 2 and Fig B in S1 Appendix). The fitted curve was overlaid with the raw sensorgrams in Fig 6A. In the second dataset, the interaction between hCGRPα and 4901 IgG was measured on a Proteon XPR36 system. In this case, the parameters for the detection flow chamber were not available. Similar parameters as in the Biacore systems, however, can still be assumed to approximate *k*_*M*_; this has been verified empirically (data not shown). As shown in Table A in S1 Appendix, the mass transport coefficient *k*_*M*_ between analyte hCGRPα and immobilized 4901 IgG was determined to be about 8.5x10^7^ RU/M/s. Finally, the active concentration and rate constants were estimated (Table 2 and Fig C in S1 Appendix) and the fitted curved were showed in Fig 6B. Overall, the fitted SPR responses based on the estimated parameters matched the observed sensorgrams very well in both cases.

As shown in Table 2, our method obtained similar dissociation rate constant *k*_*d*_ but different association rate constant *k*_*a*_ compared with the ones estimated in the original reference papers in both tests. Again, this was reasonable, since *k*_*d*_ estimation didn’t depend on the actual analyte concentration and can be determined correctly without knowing it. On the other hand, *k*_*a*_ estimation relied on the accurate information of the analyte concentration, more precisely the active concentration of the analyte. In the original papers, the total analyte concentrations were taken as a known input in the estimation. These values, however, were different from the active concentrations of analyte. In the first dataset, the active concentration was determined to be about 50% of the total concentration, and the difference between the two concentrations resulted in a higher and probably more accurate estimation of association constant *k*_*a*_. In the second dataset, the difference in *k*_*a*_ estimation came in not only due to the difference between the active concentration in our approach and the total concentration in the original, but also due to the way in which the data analysis was carried out in the original paper. In the reference paper, a Langmuir model that assumed no mass transport effect was used. Under these conditions, one expects to underestimate the kinetic rate constants. The proposed Bayesian method assumed a model with non-negligible mass transport and was thus more likely to produce more accurate estimates.

In conclusion, the proposed Bayesian approach in this work, assuming a partial mass transport-limited model and exploiting the activate analyte concentration, led to more accurate estimation of kinetic rate constants than was obtained using other methods

## Software Availability

Project name: SPR_MCMC; Projection home page: https://sourceforge.net/projects/sprmcmc/; Operating systems: Windows; Programming Language: C#; License: Free for academic use.

## Supporting Information

S1 Appendix The supporting information for the Bayesian inference approach to estimate the active concentration and affinity constants. (DOCX)
