# Supplementary material for: Bayesian Estimation of the Active Concentration and Affinity Constants Using Surface Plasmon Resonance Technology
Source: PLoS One. 2015 Jun 22;10(6):e0130812. doi: 10.1371/journal.pone.0130812 (PMC4476803; doi:10.1371/journal.pone.0130812)
Supplement: S1 Appendix — (DOCX) [file pone.0130812.s001.docx]

***Supplementary materials***

**Diffusion coefficient (D)**

In Eq. (5) in the Theory section, the analyte diffusion coefficient D is needed to estimate *h_diff_*, the characteristic height of the diffusion layer. With the measured SPR area being known, the diffusion coefficient D can be estimated by Stoke’s law and Einstein-Sutherland equation [[1](#_ENREF_1)]

where MW is the molecular mass of the analyte, $\bar{v}$ is the specific density (normally around 7.0-7.55 x 10^-4^ m^3^/kg for proteins), *k* is the Botlzman’s constant (1.381x10^-23^ J/K), *T* is the absolute temperature, *N_a_* is Avogadro’s number (6.022x10^23^ /mol). *η* is the viscosity (about 0.001 kg/(s·m)), and $\left( f | f_{0} \right)$ is the friction factor of the analyte relative to a sphere of same size (for most globular proteins a reasonable estimate of value 1.2 can be assumed) [[1](#_ENREF_1)].

**Mass transport limited binding and kinetic binding**

Eq. (7) in the Theory section can also be used to identify conditions for mass transport limited binding and for kinetic binding. A limit coefficient, *k_a_*[*B*]*/k_M_* (or more practically *k_a_*[*B_max_*]*/k_M_* where [*B_max_*] denotes the total amount of ligand on the surface), can be obtained from the equation for this purpose. When *k_a_[B]/k_M_ <<1,* the kinetic case is identified, where the mass transport flux is much higher than the heterogeneous rate constant. In this case, Eq. (7) can be simplified as

$$\frac{d[AB]}{dt}=k_{a}[A_{0}][B]-k_{d}[AB]$$

which can be analytically solved into a form known as the Langmuir model. In another limiting case, this limit coefficient is much larger than 1, *i.e.*, *k_a_*[*B*]*/k_M_ >>1,* the reaction rate is limited by mass transport and Eq. (7) has the form as

for which there is no analytical solution. By comparing binding curves calculated from Eq.(7) with different limiting coefficients, the degree of mass transport influence can be demonstrated clearly. When the limiting coefficient below 0.5, the calculated response curves are quite similar to the true kinetic curve and errors in rate constant estimation will be less than 10% [[2](#_ENREF_2), [3](#_ENREF_3)]. As the limiting coefficient increases, it is evident that mass transfer effects start to dominate the response curves. The mass transport limitation condition for a given analyte-ligand system can be changed by varying the total amount of immobilized ligand [*B_max_*] and the mass transport rate *k_M_*.

**Table A. Derivation of the mass transport coefficient *k_m_* based on the molecular weight of the analyte.**

| **Dataset** | **Analyte** | **Analyte**  **Molecular Weight**  **(kDa)** | **Diffusion Coefficient**  **(m^2^/s)** | **Mass Transport**  **coefficient** | | **Biosensor** | **Reference** |
| --- | --- | --- | --- | --- | --- | --- | --- |
|  |  |  |  | **(m/s)** | **(RU/M/s)** |  |  |
| #1 | 4-CBS | 0.201 | 4.76x10^-10^ | 5.15x10^-05^ | 1.03x10^7^ | SensiQ Pioneer | *Determined in this work.* |
| #2 | BMP10 | 48 | 7.55x10^-11^ | 1.67x10^-05^ | 8.04x10^8^ | Biacore 3000 | *Castonguay et al.* |
| #3 | hCGRPα | 3.789 | 1.78x10^-10^ | 2.24x10^-05^ | 8.5x10^7^ | Proteon XPR36 | *Abdiche et al.* |

4-CBS, 4-carboxybenzenesulfonamide; BMP10, bone morphogenetic protein 10; hCGRPα, human calcitonin gene-related peptide alpha.

The mass transport coefficient *k_m_* was derived based on the molecular weight and diffusion coefficient of the analyte according to Eqs. (9) and (10) in the main text. The measured SPR area in Biacore can be assumed to be known with the average value of an area of a length 2.0mm, a width 0.5mm situated in the middle of the flow cell [[4](#_ENREF_4)]. The height of the flow cell is 0.05mm. $l_{1}$, and $l_{2}$ in the equations are the lengths to the start and end, respectively, of the detection area from inlet of the flow cell, which are known as 0.4 and 2.0mm, respectively [[4](#_ENREF_4)]. In SensiQ Pioneer biosensors, according to the manufacturer, the dimensions of the flow cell are width=0.635mm, height=0.05mm and length=3mm. Therefore, when the molecular weight and the bulk flow rate of the analyte are also given, *k_M_* can be obtained. If the diffusion coefficient D of the analyte is not known, it can be estimated by Stoke’s law and Einstein-Sutherland equation [[1](#_ENREF_1)] as in Eq .

When the detection area lengths (*l_1_*, and *l_2_*) or other parameters (such as height (h), width (w) or length (l)) are not easily obtained for a biosensor system such as BioRad XPR system in dataset #2, *k_M_* can still be approximated using above calculation. This has been verified empirically (data not shown).

**Figure A. The trace plots for the analysis of SPR response data between CAII and 4-CBS.**

The data were generated on a SensiQ Pioneer biosensor under a partial mass-transport limited condition*.* The proposed Bayesian approach was then applied to estimate the active concentration and rate constants as well as *R_max_* and *R_0_*, based on the derived mass transport coefficient *k_m_* = 1.03x10^7^ RU/M/s (shown in Table A in S1 Appendix).

**
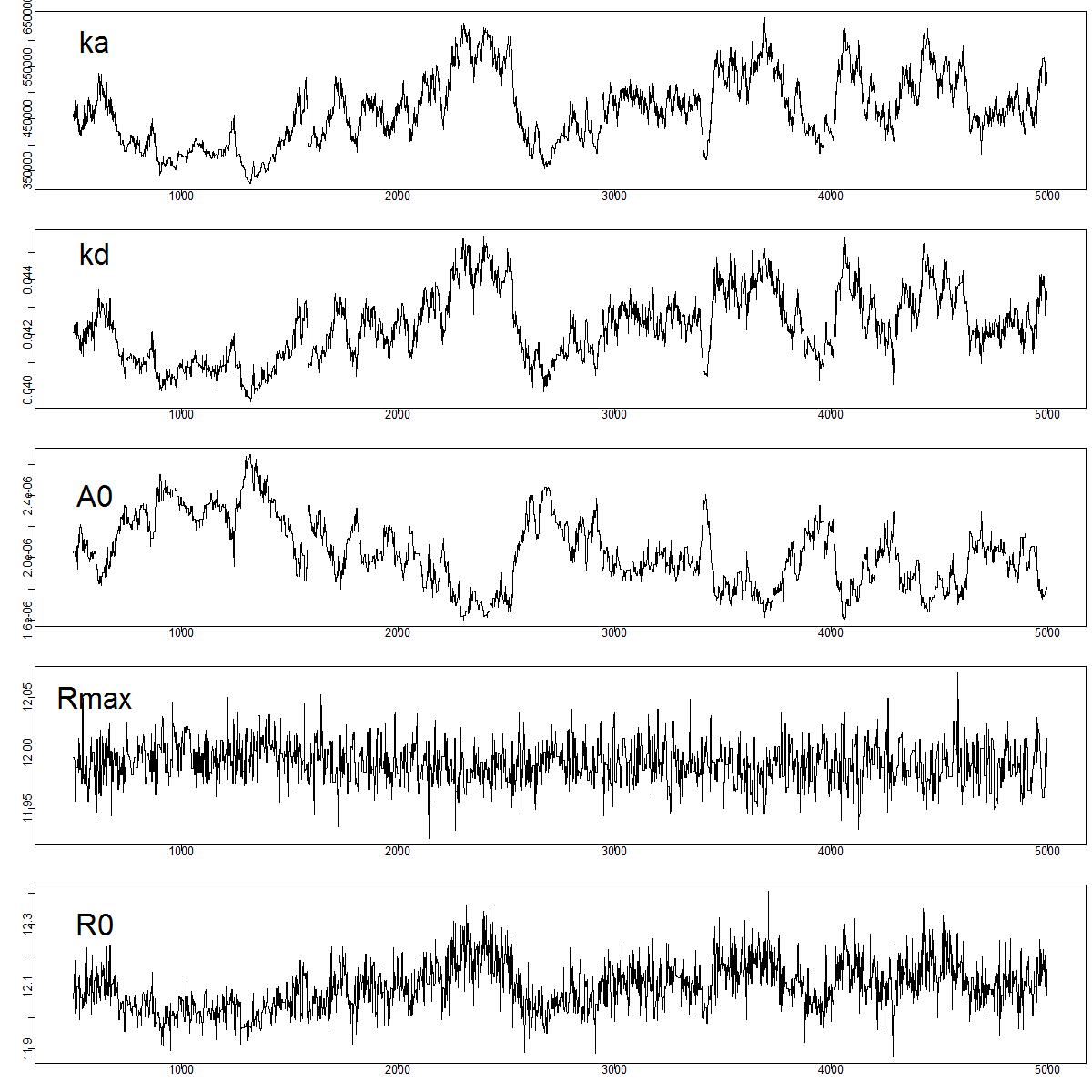
**

**Figure B. The trace plots for the analysis of SPR response data between BMP10 and hEng^ECD^-hFC.**

The data were generated on a Biacore 3000 system in the work done by Castonguay *et al* [[5](#_ENREF_5)]*.* The proposed Bayesian approach was applied to estimate the active concentration and rate constants as well as *R_max_* and *R_0_*, based on the derived mass transport coefficient *k_m_* = 8.04x10^8^ RU/M/s (shown in Table A in S1 Appendix).


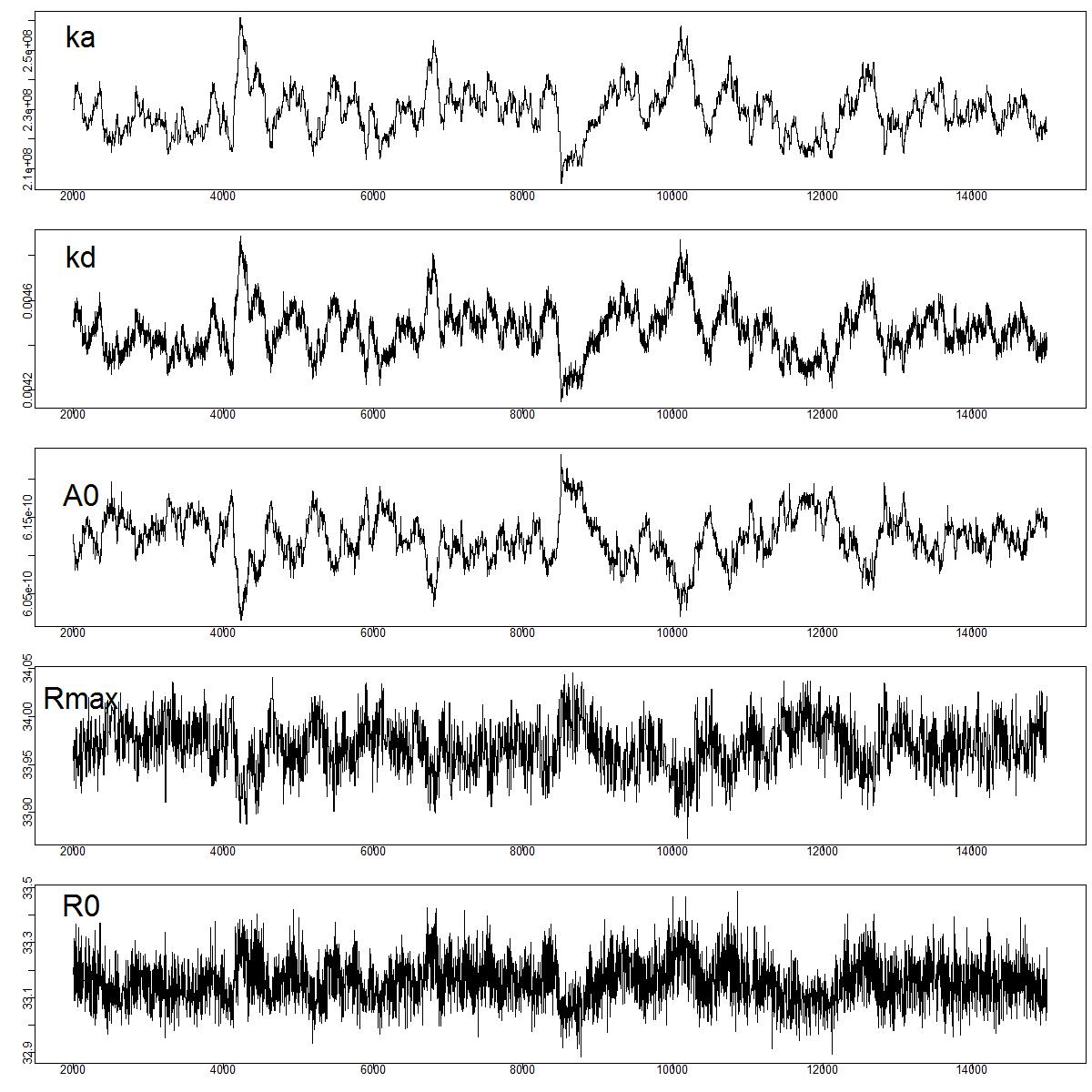


**Figure C. The trace plots for the analysis of SPR response data between hCGRPα and 4902 IgG.**

The data were generated on a Proteon XPR36 system in the work done by Abdiche *et al* [[6](#_ENREF_6)]*.* The proposed Bayesian approach was applied to estimate active concentration and rate constants as well as *R_max_* and *R_0_*, based on the derived mass transport coefficient *k_m_* = 8.5x10^7^ RU/M/s (shown in Table A in S1 Appendix).


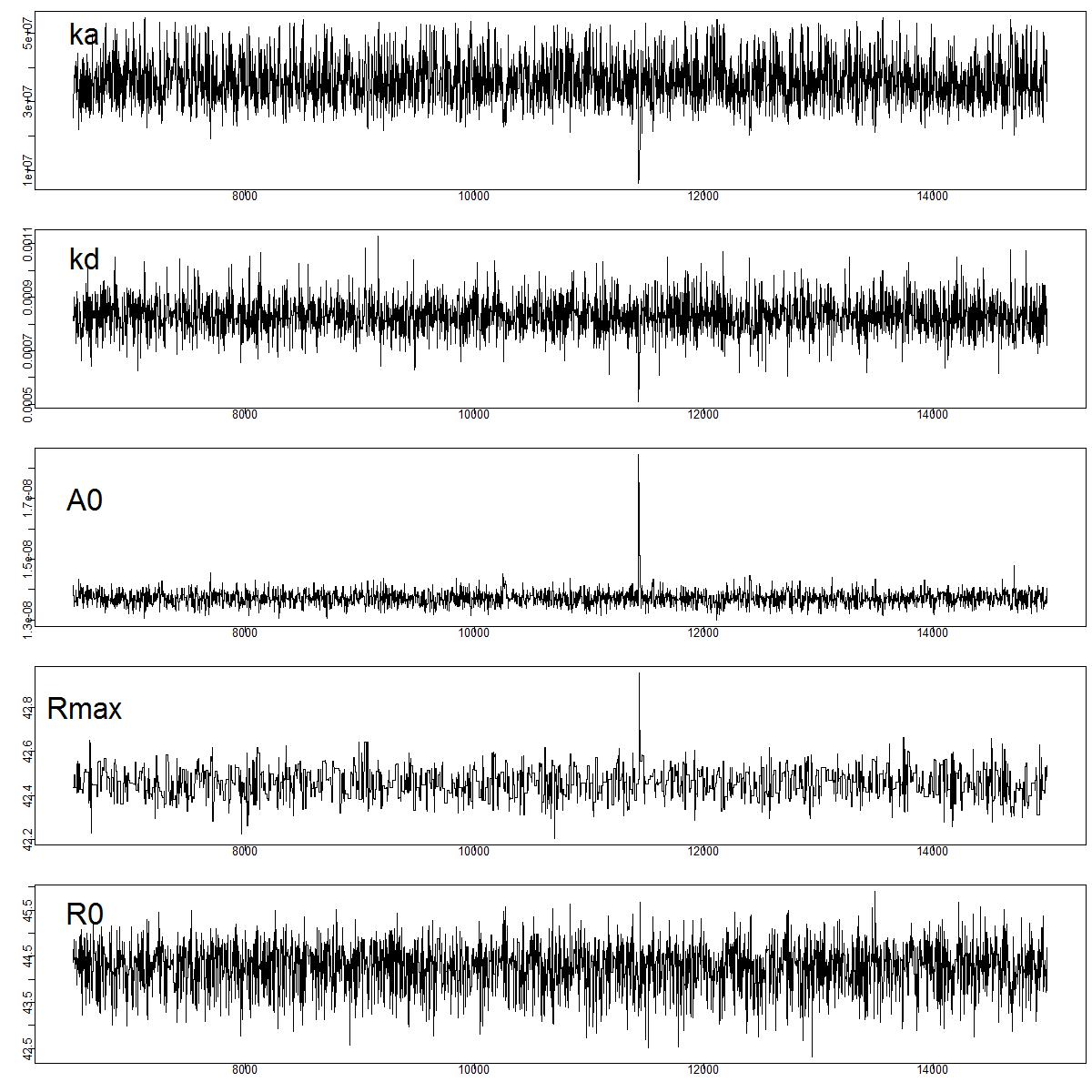


1. Knowles, P.F., *Biophysical chemistry: Part II ‘Techniques for the study of biological structure and function’: By CR Cantor and PR Schimmel. pp 503. WH Freeman and Co, Oxford. 1980. £20.70/£10.60 (paperback) ISBN 0-7167-1189-3/0-7167-1190-7 (paperback).* Biochemical Education, 1981. **9**(4): p. 157-157.

2. Glaser, R.W., *Antigen-antibody binding and mass transport by convection and diffusion to a surface: a two-dimensional computer model of binding and dissociation kinetics.* Analytical biochemistry, 1993. **213**(1): p. 152-61.

3. Karlsson, R., et al., *Kinetic and Concentration Analysis Using BIA Technology.* Methods, 1994. **6**(2): p. 99-110.

4. Christensen, L.L., *Theoretical analysis of protein concentration determination using biosensor technology under conditions of partial mass transport limitation.* Analytical biochemistry, 1997. **249**(2): p. 153-64.

5. Castonguay, R., et al., *Soluble endoglin specifically binds bone morphogenetic proteins 9 and 10 via its orphan domain, inhibits blood vessel formation, and suppresses tumor growth.* The Journal of biological chemistry, 2011. **286**(34): p. 30034-46.

6. Abdiche, Y., et al., *Determining kinetics and affinities of protein interactions using a parallel real-time label-free biosensor, the Octet.* Analytical biochemistry, 2008. **377**(2): p. 209-217.
